# Different Brain Connectivity between Responders and Nonresponders to Dual-Mode Noninvasive Brain Stimulation over Bilateral Primary Motor Cortices in Stroke Patients

**DOI:** 10.1155/2019/3826495

**Published:** 2019-04-07

**Authors:** Jungsoo Lee, Ahee Lee, Heegoo Kim, Mina Shin, Sang Moon Yun, Youngjin Jung, Won Hyuk Chang, Yun-Hee Kim

**Affiliations:** ^1^Department of Physical and Rehabilitation Medicine, Center for Prevention and Rehabilitation, Heart Vascular Stroke Institute, Samsung Medical Center, Sungkyunkwan University School of Medicine, 81 Irwon-ro, Gangnam-gu, Seoul 06351, Republic of Korea; ^2^Department of Health Sciences and Technology, Department of Medical Device Management & Research, Department of Digital Health, SAIHST, Sungkyunkwan University, 81 Irwon-ro, Gangnam-gu, Seoul 06351, Republic of Korea; ^3^Department of Global Prestige Radiological Science, Dongseo University, 47 Jurye-ro, Sasang-gu, Busan 47011, Republic of Korea

## Abstract

Noninvasive brain stimulation (NBS), such as repetitive transcranial magnetic stimulation (rTMS) or transcranial direct current stimulation (tDCS), has been used in stroke patients with motor impairment. NBS can help recovery from brain damage by modulating cortical excitability. However, the efficacy of NBS varies among individuals. To obtain insights of responsiveness to the efficacy of NBS, we investigated characteristic changes of the motor network in responders and nonresponders of NBS over the primary motor cortex (M1). A total of 21 patients with subacute stroke (13 males, mean age 59.6 ± 11.5 years) received NBS in the same manner: 1 Hz rTMS on the contralesional M1 and anodal tDCS on the ipsilesional M1. Participants were classified into responders and nonresponders based on the functional improvement of the affected upper extremity after applying NBS. Twelve age-matched healthy controls (8 males, mean age 56.1 ± 14.3 years) were also recruited. Motor networks were constructed using resting-state functional magnetic resonance imaging. M1 intrahemispheric connectivity, interhemispheric connectivity, and network efficiency were measured to investigate differences in network characteristics between groups. The motor network characteristics were found to differ between both groups. Specifically, M1 intrahemispheric connectivity in responders showed a noticeable imbalance between affected and unaffected hemispheres, which was markedly restored after NBS. The responders also showed greater interhemispheric connectivity and higher efficiency of the motor network than the nonresponders. These results may provide insight on patient-specific NBS treatment based on the brain network characteristics in neurorehabilitation of patients with stroke. This trial is registered with trial registration number NCT03390192.

## 1. Introduction

Noninvasive brain stimulation (NBS), such as repetitive transcranial magnetic stimulation (rTMS) or transcranial direct current stimulation (tDCS), has been used to facilitate the recovery of motor function in patients with stroke by modulating the excitability of intracortical neuronal circuits [1–3]. Stroke has been consistently reported in animal and human studies to disrupt interhemispheric balance and interactions [4–7]. NBS is intended to restore disrupted interhemispheric balance and to reduce transcallosal inhibition caused by stroke. In general, excitatory rTMS or anodal tDCS over the ipsilesional primary motor cortex (M1) is used to increase ipsilesional intracortical excitability, and inhibitory rTMS or cathodal tDCS over the contralesional M1 is applied to decrease overexcitability in the contralesional hemisphere [8–11]. Recently, multisite and dual-mode stimulations have been shown to effectively modulate interhemispheric imbalance of cortical excitability [12–14]. Various approaches with these techniques have been developed to improve the efficacy of NBS, which varies among individuals according to systematic reviews [15–18]. This variability may have diverse causes. Previous studies of the interindividual variability of NBS efficacy have focused on various NBS paradigms and experimental designs [16]. In addition, differences in NBS efficacy based on brain-derived neurotrophic factor (BDNF) genotype have been reported [19]. However, studies on brain connectivity in terms of differences in NBS efficacy are rare [20, 21]. NBS induces changes in connectivity between remote brain regions beyond local effects within the stimulated region [22–24]. NBS over a specific region increases or decreases ipsilateral corticocortical connectivity [25–27]. Furthermore, NBS can induce changes in the interhemispheric connectivity between bilateral hemispheres and brain network topography [24, 28, 29]. Variations in the ability of NBS to change this connectivity may depend on the properties of brain connectivity before stimulation. We hypothesized that differences in brain network balance before stimulation affect NBS efficacy. To test our hypothesis, resting-state functional magnetic resonance imaging (fMRI) was performed before and after simultaneous dual-mode stimulation (inhibitory rTMS over contralesional M1 and anodal tDCS over ipsilesional M1). To investigate network characteristics based on NBS efficacy, brain connectivity before NBS and changes in connectivity after NBS were assessed.

## 2. Materials and Methods

### 2.1. Participants and Experimental Design

Subacute stroke patients were prospectively recruited to investigate the efficacy of simultaneous dual-mode stimulation using low-frequency rTMS and anodal tDCS. Twenty-one subacute stroke patients (13 males and 8 females, mean age 59.6 ± 11.5 years) underwent dual-mode stimulation in this study. All patients received inpatient rehabilitation therapy during their participation in this experiment. Inpatient rehabilitation therapy was performed for 3 weeks which included daily physical therapy for 2 hours and occupational therapy for 1.5 hours. Speech-language therapy was also provided as needed. All patients also received medication for secondary prevention and control of hypertension or diabetes mellitus as the neurologist or internist recommended. The inclusion criteria were age over 18 years, subacute stage less than 4 weeks after stroke (16.4 ± 5.2 days, range 10-27 days), and a total Fugl-Meyer Assessment (FMA) motor score [30] under 84. The maximum possible score of FMA is 226 points. The scale includes motor score, sensory function, balance, joint range of motion, and joint pain. In this study, the FMA score corresponds to the FMA motor score which is one of the domains. The FMA motor score is a stroke-specific impairment index ranging from 0 to 100 (upper extremity 0-66, lower extremity 0-34), with a lower score indicating greater motor impairment. The exclusion criteria were a major, active, underlying neurological disease or psychiatric disease, a history of seizure, or metallic implants in the brain. Twelve age-matched healthy controls (8 males and 4 females, mean age 56.1 ± 14.3 years) were also recruited. This study was conducted in accordance with the principles of the Declaration of Helsinki. Ethical approval was obtained from the Institutional Review Board of Samsung Medical Center. All participants understood the experimental procedures and signed a consent form.

**Figure 1:**
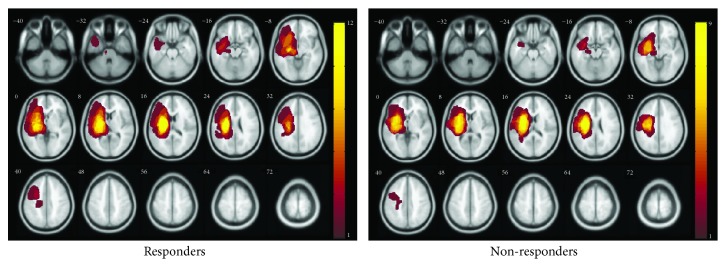
Lesion maps. Left lesions are flipped to the right hemisphere, and all lesions are overlaid on the right hemisphere. The colored bars indicate the number of patients.

All participants underwent 10 daily sessions of dual stimulation for 2 weeks. Inhibitory (1 Hz) rTMS was applied over the contralesional M1 for 20 minutes and simultaneous anodal tDCS on the ipsilesional M1. Each participant's FMA score was assessed, and resting-state fMRI was performed before stimulation (prestimulation) and 2 months after stimulation (poststimulation). Because the BDNF genotype reportedly affects motor recovery from rTMS in patients with stroke [19], blood samples from participants were assessed for the presence of the BDNF Val^66^Met polymorphism using PCR-RFLP [31]. Two patients refused the blood test; therefore, BDNF genotype was identified in 19 patients. Initial severity was measured by the National Institute of Health Stroke Scale (NIHSS) in ischemic stroke or Glasgow Coma Scale (GCS) in hemorrhagic stroke. Each patient lesion was segmented on a T1-weighted structural image. The lesion maps of all patients were made using lesion mapping software (MRIcro software http://www.cabiatl.com/mricro/mricro/index.html). The lesion was normalized into the standard MNI space. Lesion maps of both groups were visualized with the xjView toolbox (http://www.alivelearn.net/xjview) (Figure 1).

Patients were classified into two subgroups based on the functional improvement of the affected upper extremity: the responder group (7 males and 5 females, mean age 58.8 ± 13.1 years) and nonresponder group (6 males and 3 females, mean age 60.6 ± 11.3 years). The cutoff value was determined as 10 points from the minimal clinically important difference of Shelton et al.'s study [32]. The responder group had an increase in the FMA upper extremity (FMA-UE) score of 10 points or more, and the nonresponder group had an increase of less than 10 points. The clinical characteristics such as initial motor impairment, initial severity, lesion information, and numbers of patients who have BDNF Val^66^Met polymorphism did not differ between the groups. The clinical characteristics of each group are listed in Table 1.

### 2.2. Determination of the Location of the Target Area Using Single-Pulse TMS

Single-pulse TMS was performed to determine the location of M1 and to evaluate cortical excitability. All participants were seated in a reclining armchair with both hands pronated. Electromyography (EMG) electrodes were attached to record signals from the contralateral first dorsal interosseous muscle. EMG activity was amplified using a Medelec Synergy EMG/EP system (Medelec, Oxford, UK), and raw signals were passed through a bandpass filter at 10–2,000 kHz. A TMS system (Magstim Rapid2 stimulator; Magstim Ltd., Carmarthenshire, UK) and 70 mm figure-eight coil were used. The electromagnetic current flowed perpendicular to the central sulcus because the coil handle was oriented 45° posterior to the midline, as described previously [33, 34]. Using the international 10-20 system, the vertex (Cz) point was marked and the initial scalp location was identified 5 cm lateral to the intersection line from the vertex to the preauricular point. An optimal location (hot spot) with the highest MEP amplitude and shortest latency was determined by moving in 1 cm in each direction with intervals of 5 s. After the determination of the hot spot, the stimulation intensity was gradually adjusted to define the resting motor threshold (rMT) over the hot spot. The rMT was defined as the lowest magnetic intensity that evoked the EMG activity (MEP peak-to-peak amplitude ≥ 50 *μ*V) observed in 5 or more out of 10 consecutive trials. The examiner monitored muscle activity with real-time EMG to verify whether the patient was relaxed before stimulation.

### 2.3. rTMS and tDCS Application

Inhibitory rTMS was applied to the contralesional M1 area using a Magstim Rapid2 stimulator with two modules in each session. The rTMS stimulation was delivered at 1 Hz and 90% of the rMT. The rTMS intensity was maintained at a constant 90% of each participant's rMT. The hot spot and rMT were assessed before initial stimulation and remained identical throughout the trial. For patients in whom an MEP was absent in the ipsilesional hemisphere, the hot spot and rMT were measured using the mirror image of the contralesional hemisphere, as described previously [19]. The stimulation was delivered over 20 minutes and repeated 10 times over 10 daily sessions. Stimulation was applied to the contralesional M1 area by a researcher holding a figure-eight coil tangential to the skull. The rTMS protocol was based on safety guidelines for rTMS applications [10].

Anodal tDCS was applied to the ipsilesional M1 using a battery-driven DC stimulator (NeuroConn GmbH, Ilmenau, Germany) that consistently monitors electrical impedance with inhibitory rTMS stimulation. The cathodal tDCS was placed over the supraorbital area contralateral to the anodal tDCS. A constant current flow of 2 mA was delivered for 20 minutes through wet sponge electrodes (size: 7 cm × 5 cm) positioned over the ipsilesional M1 and contralesional supraorbital areas. tDCS stimulation consisted of fade-in and fade-out periods of 5 seconds to reduce discomfort.

### 2.4. Resting-State fMRI Data Acquisition

A Philips ACHIEVA® MRI scanner (Philips Medical Systems, Best, The Netherlands) operating at 3 T was used. During resting-state fMRI scans, participants were instructed to keep their eyes closed and remain motionless. Resting-state fMRI scans consisted of 100 whole-brain images collected with a T2^∗^-weighted gradient echo-planar imaging (EPI) sequence: 35 axial slices, slice thickness = 4 mm, no gap, repetition time = 3000 ms, echo time = 35 ms, flip angle = 90°, matrix size = 128 × 128, in-plane resolution = 1.72 × 1.72 mm^2^, and field of view = 220 × 220 mm^2^. T1-weighted images were also acquired for atlas transformation: 124 axial slices, slice thickness = 1.6 mm, no gap, matrix size = 512 × 512, in-plane resolution = 0.47 × 0.47 mm^2^, and field of view = 240 × 240 mm^2^.

### 2.5. Data Preprocessing

Data were sequentially preprocessed as follows: slice timing correction for different slice scan timings, spatial realignment for head motion correction, coregistration of a mean image of the fMRI images and a T1-weighted image, spatial normalization into standard template space, and spatial smoothing with a 6 mm full-width half-maximum Gaussian Kernel. Preprocessing was performed using the SPM8 package (Welcome Trust Centre for Neuroimaging, University College London, London, UK).

Several potential nuisance signals were removed to allow for linear regression of 9 parameters. The parameters included six parameters related to the rigid body transformation for motor correction, then one parameter each for white matter, ventricle, and global signals. Band-pass temporal filtering between 0.009 and 0.08 Hz was performed. Additional preprocessing steps such as nuisance regression and band-pass temporal filtering were performed using Matlab R2016a (The MathWorks, Natick, MA, USA).

## 3. Data Analysis

### 3.1. Construction of the Motor Network

Regions of interest (ROIs) in the motor network were derived from a previous study by Rehme et al. [35] who performed meta-analyses of many neuroimaging studies for the movement of the paretic upper limb in patients with stroke. We previously described the construction of a motor network with 24 ROIs in detail [36]. The 24 ROIs of the motor network are listed in Table 2.

### 3.2. Strength of Connectivity

The strength of the M1 intrahemispheric connectivity in the motor network was obtained by averaging the strength of connections between M1 and other regions in the ipsilateral hemisphere. A laterality index was calculated to investigate the balance of the M1 intrahemispheric connectivity as follows:

(1) $$\begin{array}{c} \text{LI}=\frac{\text{Ipsilesional M}1\text{ intrahemispheric}-\text{Contralesional M}1\text{ intrahemispheric}}{\text{Ipsilesional M}1\text{ intrahemispheric}+\text{Contralesional M}1\text{ intrahemispheric}}. \end{array}$$

The strength of the homotopic interhemispheric connectivity in the motor network was measured by averaging the strength of connections between homotopic regions in bilateral hemispheres. The strength of the overall interhemispheric connectivity was obtained by averaging the strength of crossed connections between hemispheres.

### 3.3. Global Network Efficiency

Global network efficiency is a graph measure of how efficiently information is exchanged in a network [37–39]. A larger value indicates easier information transfer across all regions of the network. Global network efficiency can be defined as follows [38]:

(2) $$\begin{array}{c} E_{\text{global}}=\frac{1}{n}\underset{i\in N}{\sum }\frac{\sum_{j\in N,j\neq i}{\left(d_{ij}\right)}^{-1}}{n-1}, \end{array}$$

where *n* is the number of regions and *d*_*ij*_ is the shortest path length between regions *i* and *j*. The shortest path length is the average minimum number of connections that must be traveled to move from one region to another [40].

### 3.4. Statistical Analyses

Several statistical tests were used to identify the significance of between- and within-group differences. To test the normality of the data, the Shapiro-Wilk test was performed. One-way analysis of variance (ANOVA) was performed to identify significant differences between groups (healthy control, responders, and nonresponders before stimulation). Tukey's post hoc test for multiple comparisons was also performed to compare the two groups. The respective statistic was additionally performed if there is no difference between the responders and nonresponders for multiple comparisons. Repeated-measure ANOVA was performed to investigate the differences between groups and time effects. A paired *t*-test was used to evaluate within-group differences before and after dual stimulation. A two-sample *t*-test or Wilcoxon rank sum test was performed depending on the normality or nonnormality of the data to compare homotopic connectivity results between hemispheres. Statistical analyses were performed using *anova1*, *multcompare*, *ranova*, *ttest*, *ttest2*, and *ranksum* in the statistics toolbox of Matlab R2016a. The threshold for the statistical significance was set at *p* < 0.05.

## 4. Results

**Figure 2:**
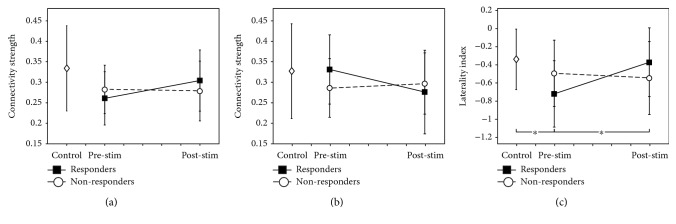
Changes in M1 intrahemispheric connectivity ((a) ipsilesional, (b) contralesional, (c) laterality index). The laterality index of the M1 intrahemispheric connectivity was significantly lower in the responder group than in the healthy control group. The laterality index in the responder group significantly increased after stimulation (^∗^*p* < 0.05).

Ipsilesional and contralesional M1 intrahemispheric connectivity in the motor network was investigated (Figures 2(a) and 2(b)). The null hypothesis of the normality test was rejected with the data in Figure 2. Several nonsignificant trends occurred in the ipsilesional and contralesional M1 intrahemispheric connectivity as follows: the ipsilesional M1 intrahemispheric connectivity in the responder group before stimulation was lower than that in the healthy controls (*p* = 0.0821). After stimulation, ipsilesional connectivity showed slight changes in the responder group (*p* = 0.0518). However, these trends were not statistically significant. The laterality index of the M1 intrahemispheric connectivity in the motor network was investigated (Figure 2(c)). The laterality index of the M1 intrahemispheric connectivity differed significantly among the healthy control, responders, and nonresponders before stimulation (*F* = 3.50, *p* = 0.0430). The laterality index in the responders was significantly lower than that in the healthy control group (*p* = 0.0344). Laterality indices in the responder and nonresponder groups were negative indicating the contralesional M1 intrahemispheric connectivity was dominant in both groups. Changes in the laterality index of the M1 intrahemispheric connectivity in the motor network were investigated. The laterality index increased toward zero after stimulation in the responder group (*p* = 0.0457) but was unchanged in the nonresponder group. Changes in the laterality index of the M1 intrahemispheric connectivity in the responder and nonresponder groups did not differ significantly (group^∗^time interactions: *p* = 0.0698).

**Figure 3:**
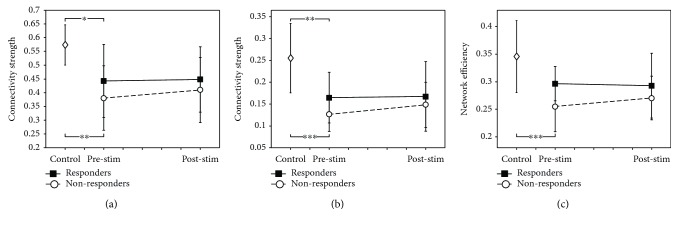
Changes in interhemispheric connectivity ((a) homotopic and (b) overall) and global network efficiency (c) of the motor network. Interhemispheric connectivity was significantly lower in the responder and nonresponder groups than in the healthy control group. Network efficiency was significantly lower in the nonresponder group than in the healthy control group (^∗^*p* < 0.05, ^∗∗^*p* < 0.01, and ^∗∗∗^*p* < 0.001, respectively).

Interhemispheric connectivity in the motor network was investigated (Figures 3(a) and 3(b)). The null hypothesis of the normality test was rejected with the data in Figure 3. Interhemispheric connectivity differed between the healthy controls and patients with stroke before stimulation (homotopic, *F* = 8.68, *p* = 0.0011; overall, *F* = 12.01, *p* < 0.001). The responder and nonresponder groups showed lower interhemispheric connectivity than the healthy control group (homotopic: responders, *p* = 0.0177, nonresponders, *p* = 0.0011; overall: responders, *p* = 0.0038, nonresponders, *p* < 0.001). The mean difference in interhemispheric connectivity between the responder group and healthy controls (homotopic, 0.1308; overall, 0.0905) was lower than that between the nonresponder group and healthy controls (homotopic, 0.1930; overall, 0.1289). Interhemispheric connectivity did not change within the groups (homotopic: responders, *p* = 0.9070, nonresponders, *p* = 0.3095; overall: responders, *p* = 0.9015, nonresponders, *p* = 0.2350), and changes between the groups did not differ after stimulation (homotopic, group^∗^time interactions: *p* = 0.6767; overall, group^∗^time interactions: *p* = 0.5309). Global network efficiency of the motor network was investigated (Figure 3(c)). Network efficiency differed significantly between the healthy control and patient groups before stimulation (*F* = 8.70, *p* = 0.001). The global network efficiency in the nonresponder group was significantly lower than that in the healthy control group (*p* < 0.001), and the value also differed between the responder and nonresponder groups from the respective statistic between both groups (*p* = 0.0230). In the respective statistic, other measures such as M1 intrahemispheric connectivity, laterality index, and interhemispheric connectivity were not significantly different between the responder and nonresponder groups prior to stimulation. Network efficiencies did not change within the groups (responders, *p* = 0.8316; nonresponders, *p* = 0.3707), and changes between groups did not differ after stimulation (group^∗^time interactions: *p* = 0.4379).

**Figure 4:**
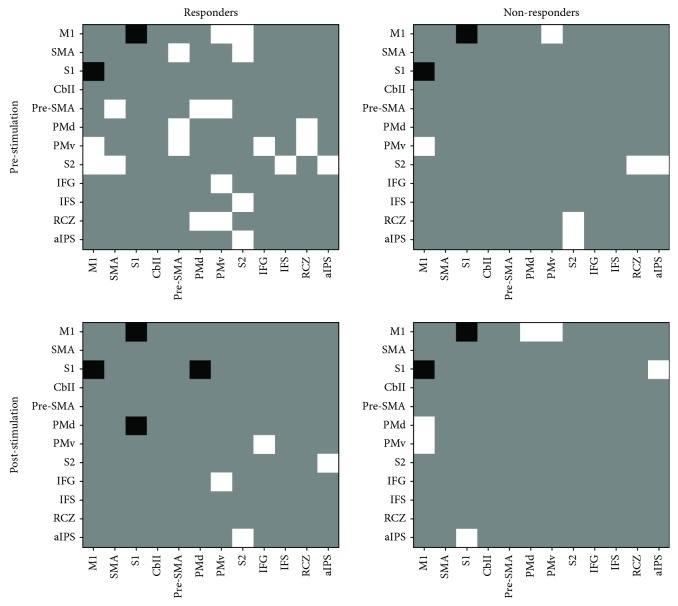
The significant difference of homotopic connectivity between hemispheres before and after stimulation. White connectivity indicates that the strength of contralesional connectivity is significantly greater than that of ipsilesional connectivity. Black connectivity indicates that the strength of ipsilesional connectivity is significantly greater than that of contralesional connectivity. Gray connectivity indicates that there is no difference in the strength of homotopic connectivity between hemispheres. The more white connectivity the adjacency matrix has, the more contralesional dominance the motor network is. The motor network in the responders before stimulation showed contralesional dominance. After stimulation in the responders, the motor network became symmetric by changing from white connectivity to gray connectivity, whereas the motor network in the nonresponders before stimulation showed relatively symmetric. After stimulation in the nonresponders, there was no change in the degree of symmetry of the motor network.

Differences in homotopic connectivity between hemispheres were investigated before and after stimulation to examine changes in the symmetry of local connectivity (Figure 4). Many connections showed contralesional dominance in the responder group before stimulation. However, the nonresponder group did not have noticeable one-sided dominance before stimulation. After stimulation, contralesional dominance in the responder group disappeared and differed from that in the nonresponder group which did not show distinct differences before or after stimulation.

## 5. Discussion

According to previous systematic reviews [15–18], the efficacy of NBS varies among individuals. The interindividual variability may have diverse causes such as NBS paradigms and experimental designs. It is hard to expect sufficient efficacy of all patients through applying common dose and a target area in patients with various characteristics. Each patient has different brain anatomy, age, lesion location, a baseline of function, neurochemistry, and genetics. These individual factors can contribute to the variability of the NBS efficacy [19, 41–45]. These studies are important in terms of providing evidence about individual NBS strategy.

In our study, causes of NBS efficacy were investigated by analyzing brain network characteristics obtained from resting-state fMRI. Motor network characteristics differed between the responder and nonresponder groups. The balance of M1 intrahemispheric connectivity and local connectivity between bilateral hemispheres showed contralesional dominance in the responder group before stimulation. This increased involvement of contralesional M1 has been reported previously [46–48]. This characteristic was noticeable in the brain connectivity of the responder group, while the contralesional M1 had less involvement in the nonresponder group. Interhemispheric connectivity and network efficiency of the initial motor network in the responder group tended to be greater than those in the nonresponder group. These results indicate that stroke patients with a disrupted network balance (contralesional dominance) who have relatively well-preserved interhemispheric connectivity and efficient network structure can expect efficacy from dual-mode stimulation.

Changes in brain connectivity induced by NBS, such as rTMS and tDCS, over the M1 area have been reported in previous studies [9, 22, 23, 49, 50]. The changes vary among studies; however, NBS over the M1 area mostly induced connectivity between the stimulated area and remote areas and particularly changes intrahemispheric connectivity [9, 23, 49]. In our study, the M1 intrahemispheric connectivity in the responder group effectively increased connectivity in the ipsilesional hemisphere and decreased connectivity in the contralesional hemisphere. An imbalanced initial motor network in the responder group might be more suitable for the purpose of the NBS which is to restore network balance.

Interhemispheric connectivity and network efficiency are important measures of brain function [4, 5, 51–53]. These two measures were relatively higher in the responder group than in the nonresponder group in this study. Higher interhemispheric connectivity and network efficiency are favorable for motor network communication between hemispheres and throughout networks. The effects of NBS which is to reduce transcallosal inhibition may be enhanced in the brains with high interhemispheric connectivity and network efficiency. The results of the previous study [36] showed neither measure significantly changed in stroke patients with severe impairment while these measures showed changes in moderately impaired patients. In this study, most participants had a severe motor impairment at inclusion; therefore, neither measure showed significant change over time.

This study had some limitations. We did not compare the damage of structural connectivity between the two groups by using diffusion tensor imaging data. Structural injury may be related to functional connectivity [54, 55]. However, we addressed other data that can compare the severity of structural damage such as lesion map, lesion volume and location, initial severity of stroke, and initial rMT. There were no significant differences in these data between the two groups which suggest that the degree of structural damage might not be different. Furthermore, the causes of the differences in network balance, interhemispheric connectivity, and network efficiency after stroke were not determined in this study. The network characteristics of responders or nonresponders in response to diverse NBS protocols were also not fully explored. Despite these limitations, our study may have a value in terms of the identification of factors related to the variability of the efficacy of NBS through brain connectivity analysis.

## 6. Conclusions

The responder group showed a disrupted balance of the M1 intrahemispheric connectivity with the contralesional hemisphere being dominant, but interhemispheric interaction and network efficiency are relatively preserved compared to the nonresponder group. These results may provide meaningful information on an individually-tailored NBS treatment by investigating brain network characteristics before stimulation and also give insights into neuroimaging biomarkers for NBS effects in restoring neural network balance of patients with stroke.

## Figures and Tables

**Table 1:** Patient characteristics.

Group	Responders	Nonresponder
Age (years)		
Mean ± SD	58.8 ± 13.1	60.6 ± 11.3
Sex (*n*)		
Male	7	6
Female	5	3
Type of stroke (*n*)		
Hemorrhagic	7	5
Ischemic	5	4
Initial stroke severity (mean ± SD)		
NIHSS (ischemic stroke)	5.6 ± 2.7	6.0 ± 1.8
GCS (hemorrhagic stroke)	13.6 ± 1.9	13.4 ± 1.7
Lesion side (*n*)		
Right	9	7
Left	3	2
Bilateral	0	0
Lesion location (*n*)		
Supratentorial	11	9
Infratentorial	1	0
Lesion volume (ml)		
Mean ± SD	51.3 ± 43.2	51.1 ± 24.3
FMA-UE scores (0-66 points) (mean ± SD)		
Prestimulation	17.8 ± 16.3	27.1 ± 25.7
Poststimulation	39.9 ± 17.1	31.2 ± 24.5
BDNF genotype		
Val/Val	1	1
Met allele	10	7
N/A	1	1
Initial rMT (%)		
Mean ± SD	82.4 ± 22.7	75.3 ± 29.4

SD: standard deviation; NIHSS: National Institutes of Health Stroke Scale; GCS: Glasgow Coma Scale; FMA-UE: Fugl-Meyer assessment upper extremity; BDNF: brain-derived neurotrophic factor; N/A: not available; rMT: resting motor threshold.

**Table 2:** ROIs in the motor networks.

No.	Region	Side	MNI coordinates		
			*x*	*y*	*z*
1	Precentral gyrus (M1)	IL	-38	-24	58
2	Precentral gyrus (M1)	CL	42	-14	52
3	Medial superior frontal gyrus (SMA)	IL	-4	-6	54
4	Medial superior frontal gyrus (SMA)	CL	4	-6	54
5	Postcentral gyrus (S1)	IL	-36	-30	60
6	Postcentral gyrus (S1)	CL	40	-28	52
7	Cerebellum (Cbll)	IL	-24	-60	-22
8	Cerebellum (Cbll)	CL	20	-50	-22
9	Medial superior frontal gyrus (pre-SMA)	IL	-2	6	54
10	Medial superior frontal gyrus (pre-SMA)	CL	2	2	56
11	Dorsolateral precentral gyrus/sulcus (PMd)	IL	-42	-10	58
12	Dorsolateral precentral gyrus/sulcus (PMd)	CL	42	-6	56
13	Ventrolateral precentral gyrus/sulcus (PMv)	IL	-46	-10	48
14	Ventrolateral precentral gyrus/sulcus (PMv)	CL	42	-6	48
15	Parietal operculum (S2)	IL	-48	-18	22
16	Parietal operculum (S2)	CL	50	-28	28
17	Inferior frontal gyrus (IFG)	IL	-48	6	6
18	Inferior frontal gyrus (IFG)	CL	48	6	6
19	Inferior frontal sulcus (IFS)	IL	-50	8	34
20	Inferior frontal sulcus (IFS)	CL	50	8	34
21	Rostral cingulate zone (RCZ)	IL	-8	14	36
22	Rostral cingulate zone (RCZ)	CL	8	14	36
23	Anterior intraparietal sulcus (aIPS)	IL	-42	-40	50
24	Anterior intraparietal sulcus (aIPS)	CL	42	-40	50

ROIs: regions of interest; IL: ipsilesional side; CL: contralesional side.

## Data Availability

The data used to support the findings of this study are restricted by the IRB of Samsung Medical Center in order to protect patient privacy. Data are available from the corresponding author Prof. Yun-Hee Kim (yun1225.kim@samsung.com) for researchers who meet the criteria for access to confidential data.
